# Exposure to Intimate Partner Violence and Hypertension Outcomes among Young Women in South Africa

**DOI:** 10.1155/2021/5519356

**Published:** 2021-04-01

**Authors:** Nicole De Wet-Billings, Motlatso Godongwana

**Affiliations:** Demography and Population Studies, Schools of Social Sciences and Public Health, University of the Witwatersrand, Johannesburg, South Africa

## Abstract

Hypertension and intimate partner violence is affecting longevity and quality of life among women worldwide. In this study, intimate partner violence is identified as a risk factor for hypertension outcomes among young women in South Africa. Using a nationally representative sample of 216 (N) young women (15–34 years old) from the South African Demographic and Health Survey, this study uses cross-tabulations and logistic regression methods to identify the odds of hypertension outcomes. Results show that between 20 and 41% of 15–34-year-old women have hypertension. Further, 68% of women with hypertension experienced physical intimate partner violence. Finally, the odds of hypertension are increased if young women experience physical (OR: 4.07; CI: 1.04726–15.82438) or sexual (OR: 2.56; CI: 1.18198–5.55834) intimate partner violence. Efforts to reduce hypertension outcomes in the country should include intimate partner violence awareness and assistance.

## 1. Introduction

Intimate partner violence is a global pandemic affecting the lives of thousands of women. Intimate partner and gender-based violence in South Africa is currently higher than it is has ever been [1, 2]. The most severe consequence of intimate partner violence is death. Globally, 30,000 women were intentionally killed by their intimate partners in 2018, a marked increase from the estimates 12,000 in 2012 [3]. In South Africa, 57.1% of female homicide deaths are by an intimate partner [4]. Recently, it has been reported that femicide is five times higher in South Africa than the global average [2]. Further, as many as 51% of South African women have experienced violence at the hands of an intimate partner in their lifetimes [2].

Intimate partner violence is also a known stressor for women, causing emotional and psychological trauma. Victims of intimate partner violence are reported to experience higher levels of depression, anxiety, and other mental health diseases [5–7]. In South Africa, a study showed that 30% of women who reported physical and emotional intimate partner abuse by their partners reported suicidal ideations [8]. Many global studies have similarly found a relationship between intimate partner violence and suicidal ideations and behaviours of victims [9–13]. Further intimate partner abuse increases the risk of sexually transmitted diseases (STDs) including HIV/AIDS transmission, among women who experience physical and sexual assault [14, 15]. Among young women (education grades 7–12) who experienced intimate partner violence in the USA, 7.1% had reported having an STD [16]. Women who are abused by their partners also suffer from chronic diseases. A study of abused women in Spain found that 36.2% reported having a chronic disease (from a range of questions asked by the researchers) and 10.1% had asthma [17]. In addition, physical injuries to victims of violence from their partners result in periods of hospitalisation and chronic pain conditions [18–21]. While this description of consequences includes various health outcomes, the list is not extensive and there is need to examine other health consequences associated with intimate partner violence.

Hypertension is a form of cardiovascular disease that is more prevalent among females than males [22, 23]. In South Africa, an estimated 34.7% of females over the age of 15 years old are diagnosed with hypertension [24]. In the past, this disease was most prevalent among older females, but recent research in the country shows that 13.7% of 15–19-year-olds and 12.5% of 20–24-year-olds have been diagnosed [24, 25]. This is most concerning since hypertension is a chronic disease which requires management through the use of medication, diet, and stress control, all of which have monetary implications which adolescents and young adults cannot afford [26].

Existing studies have identified tobacco and alcohol use, overweight and obesity, and diabetes, among others as risk factors for hypertension [27, 28]. Psychosocial factors are being investigated as contributing to cardiovascular diseases, such as hypertension. Recent studies have found that stress, depression, and social isolation contribute to cardiovascular disease risk [29–31]. A few studies have even examined the role of intimate partner violence on hypertension outcomes among women [32, 33]. However, these studies were done on countries with lower intimate partner violence prevalence than South Africa. Also, from the above, hypertension prevalence among women in South Africa is also high and increasing among young women. South Africa was selected as a country of analysis because of the high burden of intimate partner violence (62%) and hypertension among women (26.1%). This makes South Africa the ideal study site to examine the relationship between intimate partner violence and hypertension outcomes. Further, empirical evidence is needed from less-developed countries to inform health policies and programmes and update current knowledge on the potential risk factors for cardiovascular diseases, such as hypertension.

## 2. Materials and Methods

### 2.1. Data

This study utilized secondary data from the 2016 South African Demographic and Health Survey (DHS). South Africa was selected as a country of analysis because of the high burden of intimate partner violence (62%) and hypertension among women (26.1%). Permission to use the data was obtained from the DHS program. The data include variables on intimidate partner violence such as experiences of physical, emotional, and sexual violence as well as information on hypertension diagnosis. Information on the demographic, socioeconomic, and HIV status is also included. The DHS program collects data at an individual and household level. To obtain all of the variables of interest, the individual female data file, the household data file, and the HIV biomarker data were all merged into a single file [34].

### 2.2. Inclusion Criteria and Sample Size

A total of 3, 514 women (15–49 years) were interviewed in the domestic violence (DV) module of the DHS survey. Due to literature showing an increase in hypertension among young women and the need to prevent chronic illness into later adulthood, only females aged 15–34 years' old who participated in the domestic violence DV module were included. A weighted sample of 687 women responded to questions on hypertension diagnosis. The study uses the DHS survey weights and details of the survey's procedure can be found in the South African DHS report [34]. Of these females, only 605 provided responses to questions on intimate partner violence (IPV). As such, a total of 605 females (15–34 years) were analysed. Of these, 35.7% (*N* = 216) had hypertension. All missing observations were dropped from the analysis.

### 2.3. Study Variables

The outcome variable in this study is hypertension status. The question asked to respondents to obtain this variable was “Were you told that you had hypertension or blood pressure.” This variable was treated as a binary variable as it consists of two categories coded as “no” (0) and “yes” (1).

The main predictor variable was exposure to intimate partner violence. To obtain this variable, the study used proxy measures for experiences of physical violence which included experience of being pushed, shook, or had something thrown at you (no (1) or yes (2)), kicked or dragged (no (1) or yes (2)), strangled or burnt (no (1) or yes (2)), and ever been threatened with a knife, gun, or other weapon (:no (1) or yes (2)). Other IPV variables that were used were experience of emotional violence (no (1) or yes (2)), experience of sexual violence (no (1) or yes (2)), experience of both physical and sexual violence (no (1) or yes (2)), experience of physical, emotional, and sexual violence (no (1) or yes (2)).

The demographic control variables that were included in this study were as follows: age grouped in 5-year intervals as 15–19; 20–24; 25–29; and 30–34 and race (Black (1); White/Indian (2); Coloured (3). The White and Indian population groups were combined into one category because each individual category had very few weighted observations (0.95 and 1,04, respectively). Other variables included were place of residence (urban (1); rural (2)), marital status (never married (1); married (2); separated/divorced/widow (3)) and whether the participant has ever had children (no (1) or yes (2)). The socioeconomic control variables that were used were highest education level obtained (no education (0); primary (1); secondary (2); higher (3)), wealth status (poor (1); middle (2); rich (3)), employment status (unemployed (1) or employed (2)) and whether the participant received a government/social grant (no (1) or yes (2)). Lastly, the HIV status (negative (1) or positive (2)) variable was also included in the study. While the variable selection may include covariates with no direct relationship to hypertension, these variables serve to create a profile of the demographic and socioeconomic characteristics of South African women. In doing so, these characteristics encapsulate important characteristics of the population who experience intimate partner violence and are at risk of hypertension and other noncommunicable diseases.

### 2.4. Data Analysis

The data were analysed using version 14.0 of the Stata software. This study begins with providing a description of the characteristics of women with hypertension. The study describes the respondent's experiences of IPV and the demographic and socioeconomic characteristics of women with hypertension. For the inferential statistical analysis, multivariate logistic regression was used to test and show the association between IPV and hypertension as well as the influence of each of the other predictor variables on hypertension diagnosis. The results from this analysis are reported using odds ratios and confidence intervals. The level of significance is set at 0.05.

## 3. Results

**Figure 1:**
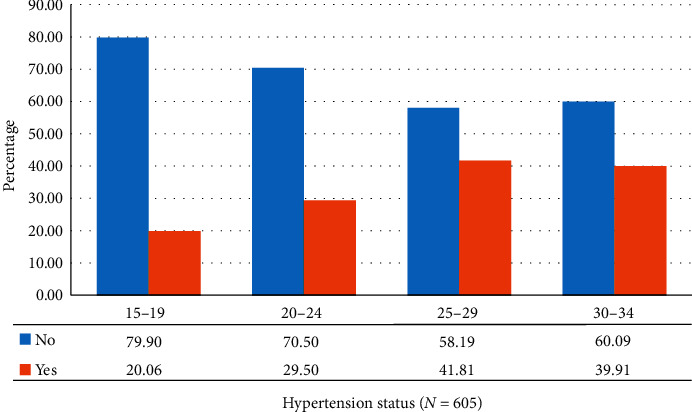
Percentage distribution of hypertension status (yes/no) by age group of the respondents, South Africa, 2016.

From figure 1, the prevalence of hypertension was highest among older females as compared to younger females. About 41.8% and 39.9% of females aged 25–29 and 30–34 years have hypertension, respectively.

**Figure 2:**
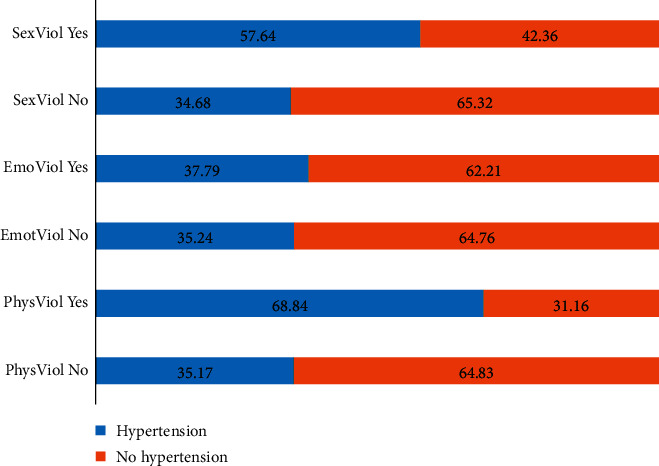
Percentage distribution of hypertension females (15–34 years old) by experiences of intimate partner violence, South Africa, 2016.

Figure 2 shows that 68.84% of the women who are hypertensive have experienced physical violence (PhysViol Yes) from their partners. Also, 37.39% of hypertensive women have experienced emotional violence (EmoViol Yes) and 57.64% experienced sexual violence (SexViol Yes).

In terms of the demographic characteristics (Table 1), the levels of hypertension diagnosis were higher among coloured South Africans (46.4%), females that resided on rural areas (37.9%), and females who reported to have at least one child (40.3%). About the socioeconomic characteristics, hypertension was prevalent among females who were married as compared to those that have never married or were separated. For example, almost 40% of females aged 15–34 years who were married were hypertensive. Females with no education (42.6%) and with a poor wealth status (38.6%) had higher levels of hypertension, respectively. Only about 40% of females who were employed had hypertension. However, the level of hypertension was higher among females who received a social grant compared to those that did not. Lastly, hypertension was not as prevalent among HIV positive females in this study. Only about 30.8% of females living with HIV were diagnosed with hypertension which is less than the percentage (36.8) of HIV negative females that had hypertension in this study. Only about 37.7% of females who experienced a combination of physical, sexual, and emotional violence had hypertension.

In the unadjusted logistic regression model (Table 2), physical violence was significantly associated with hypertension diagnosis. For example, females who had experienced some form of physical violence were 4.07 (*P* < 0.05) times more likely to be diagnosed with hypertension compared to those that have never experienced any form of violence [CI: 1.04726–15.82438]. The odds of hypertension were significantly higher among females who experienced sexual violence compared to those that have not [OR: 2.56; CI: 1.18198–5.55834].

Concerning the respondent's demographic characteristics, the odds of hypertension were significantly higher among females aged 25–29 [OR: 2.8621; CI: 1.439–5.69562] and 30–34 [OR: 2.64; CI: 1.32358–5.28812], respectively. A significant association was found between children ever born and hypertension status. The odds of hypertension diagnosis were 2.55 times (*P* < 0.05) among females who reported to have at least one child as compared to those that have no children [1.64351–3.98327]. Additionally, receipt of social grant was a significant predictor of hypertension status. For example, females who received a social grant were 3.33 (*P* < 0.05) times more likely to be diagnosed with hypertension [CI: 2.30984–4.82248].

## 4. Discussion

Hypertension is a chronic disease that compromises survival and quality of health outcomes. Females are disproportionately affected by hypertension globally and in South Africa [22, 35, 36]. While efforts, such as the South African Hypertension Society (https://www.hypertension.org.za/) which supports the dissemination of information and blood pressure testing throughout the country, are in place, intimate partner violence may affect women's ability to access care and treatment. This study was done to identify the relationship between intimate partner violence and hypertension outcomes among young women in South Africa. Results address pertinent risk factors for hypertension among young women in a country where intimate partner violence and gender-based violence have reached epidemic proportions.

This study finds an association between intimate partner violence and hypertension among young women in South Africa. Other studies have similarly found a relationship between gender-based and intimate partner violence and chronic health conditions, including hypertension [17, 32, 33, 37]. A confounding factor may be the stress caused by intimate partner violence that is also a contributor to hypertension [33, 37]. Moreover, victims of intimate partner violence are known to also have less control over their finances, little or no decision-making authority within households, and as a consequence are unable to seek medical healthcare [38–40]. Under these circumstances, it is difficult to prevent or get treatment for a range of medical conditions, including hypertension. It is recommended that young women at risk of hypertension manage their diet, avoid stress, and seek medical attention as soon as possible [41]. However, with experiences of physical, sexual, and emotional violence from partners, these young women might not prioritise prevention or treatment measures or may be denied support from their abusive partners.

Hypertension also increases with age among women in South Africa. Age is a known risk factor for hypertension outcomes [42], but this study shows a significant proportion of youth who are at risk of the disease. Studies have identified that diet, alcohol in-take, sedentary lifestyles, and pregnancy are risk factors for hypertension in young women [27, 43]. In this study, 76% of respondents had ever given birth. Results here also show that having ever given birth is associated with increased risk of hypertension. Young mothers in South Africa also have high rates of intimate partner violence [44, 45]. One study of women who gave birth at a Durban hospital found that nearly a quarter of women experienced intimate partner violence in the first nine months postpartum [44]. The stress of experiencing abuse postpartum could exacerbate the development of hypertension. Therefore, more efforts should be made to assist these young women.

This study uses secondary data, and therefore it cannot be determined if the intimate partner violence experiences predate the development of hypertension in young women in South Africa. Second, from the data, it is not possible to measure stress levels and other possible confounding factors that contribute to hypertension outcomes. Further, the study cannot determine if the hypertension status of these young women was pregnancy-related, as is the case of pre or postpartum hypertension. In addition, from the data, it is not possible to determine if the ways in which young women who experience intimate partner violence and have hypertension are managing their chronic illness. This would have offered insight into whether their abuse is in fact a hindrance to the care they receive for hypertension. Finally, the study is based on self-reporting of hypertension status and not blood pressure measurements producing a reporting bias. However, in the absence of survey data which collects blood pressure and other vital measurements, self-reporting of disease diagnosis provides sufficient data, but a study that examines blood pressure measurements, in addition to self-reporting, would enhance our understanding in this area.

There are a few strengths of this study. First, the study uses a population-based sample where other studies have used smaller samples of women from clinics or enrolled in site-specific studies. Second, the study addresses two prevalent and growing issues of young women in South Africa, hypertension and intimate partner violence. Both are known to have detrimental consequences for women, and this study shows that there is a positive relationship between the two.

## 5. Conclusions

Intimate partner violence is associated with hypertension. Stress caused by violence perpetrated by a spouse or partner could be a confounding factor in the development of hypertension in young women. As a chronic illness which requires medication and specialised dietary requirements among others, hypertension is a costly disease. Young women in South Africa (and globally) are not all financially independent and cannot bear the costs of management and treatment alone. Therefore, all efforts to prevent the development of hypertension in young people should be pursued. For young women who experience intimate partner violence, this study shows that efforts to eradicate abuse will also aid in the reduction of hypertension. Identifying that intimate partner violence is a risk factor for hypertension assists local programmes and policies to develop more holistic approaches to care, prevention, and management strategies for young women.

Future research should focus on causality and determine if intimate partner violence does result in later hypertension outcomes. Further, a qualitative study which examines the coping mechanisms of intimate partner violence victims in relation to the management of hypertension will inform direct ways medical and social programmes can provide assistance to these women.

## Figures and Tables

**Table 1:** Frequency and percentage distribution of respondents' characteristics by hypertension status, South Africa, 2016.

Respondent characteristics	Total	Hypertension		No hypertension	
	N	N	%	N	%
Total	605	216	35.70	389	64.30

*Race*					
** **Black	552	195	35.28	357	64.72
** **White/Indian	12	2	18.85	10	81.15
** **Coloured	42	19	46.43	22	53.57

*Place of residence*					
** **Urban	421	146	34.74	275	65.26
** **Rural	184	70	37.97	114	62.03

*Children ever born*					
** **No	144	30	20.91	114	79.09
** **Yes	461	186	40.35	275	59.65

*Marital status*					
** **Never married	391	135	34.65	255	65.35
** **Married	196	78	39.77	118	60.23
** **Separated	18	3	15.35	16	84.65

*Education level*					
** **No education	5	2	42.66	3	57.34
** **Primary	41	10	25.08	30	74.92
** **Secondary	504	187	37.02	318	62.98
** **Higher	55	17	30.97	38	69.03

*Wealth status*					
** **Poor	246	95	38.61	151	61.39
** **Middle	159	59	37.09	100	62.91
** **Rich	200	62	31.07	138	68.93

*Employment status*					
** **Unemployed	418	141	33.83	276	66.17
** **Employed	188	75	39.94	113	60.06

*Social grant status*					
** **No	434	120	27.7	314	72.3
** **Yes	171	96	56.12	75	43.88

*HIV*					
** **Negative	493	182	36.82	312	63.18
** **Positive	112	35	30.87	77	69.13

*Physical*, *sexual*, *and emotional violence*					
** **No	490	173	35.24	318	64.76
** **Yes	115	43	37.79	71	62.21

**Table 2:** Unadjusted logistic regression of the odds of hypertension by characteristics of the respondents.

Respondent characteristics	Odds ratio	*P* value	Confidence interval
*IPV*			
Physical violence			
** **No	RC		
** **Yes	4.07	0.043*∗*	1.04726–15.82438

Emotional violence			
** **No	RC		
** **Yes	1.11	0.608	0.73334–1.69947

Sexual violence			
** **No	RC		
** **Yes	2.56	0.017*∗*	1.18198–5.55834

** **Physical, sexual, and emotional violence			
** **No	RC		
** **Yes	1.11	0.608	0.73334–1.69947

*Demographic*			
** **Age			
** **15–19	RC		
** **20–24	1.66	0.159	0.81858–3.39567
** **25–29	2.86	0.003*∗*	1.43921–5.69562
** **30–34	2.64	0.006*∗*	1.32358–5.28812

** **Race			
** **Black	RC		
** **White/Indian	0.42	0.250	0.09974–1.82041
** **Coloured	1.58	0.152	0.84344–2.99638

** **Place of residence			
** **Urban	RC		
** **Rural	1.14	0.447	0.80288–1.64594

** **Children ever born			
** **No	RC		
** **Yes	2.55	0.000*∗*	1.64351–3.98327

*Socioeconomic status*			
** **Marital status			
** **Never married	RC		
** **Married	1.24	0.225	0.87388–1.77345
** **Separated	0.34	0.101	0.09474–1.23430

** **Highest level of education obtained			
** **No education	RC		
** **Primary	0.45	0.402	0.06941–2.91762
** **Secondary	0.79	0.791	0.13888–4.4971
** **Higher	0.60	0.586	0.09758–3.72841

** **Wealth status			
** **Poor	RC		
** **Middle	0.93	0.759	0.62147–1.41480
** **Rich	0.71	0.098	0.48317–1.06355

** **Employment status			
** **Unemployed	RC		
** **Employed	1.30	0.147	0.91184–1.85675

** **Social grant status			
** **No	RC		
** **Yes	3.33	0.000*∗*	2.30984–4.82248

** **HIV status			
** **Negative	RC		
** **Positive	0.76	0.236	0.49295–1.19032

*∗*Significant (*P* < 0.05).

## Data Availability

The data used in this study are from the South African Demographic and Health Survey (SADHS) and can be downloaded for analysis from https://dhsprogram.com/.
